# Serum tryptase detected during acute coronary syndrome is significantly related to the development of major adverse cardiovascular events after 2 years

**DOI:** 10.1186/s12948-015-0013-0

**Published:** 2015-06-02

**Authors:** Elide Anna Pastorello, Laura Farioli, Laura Michelina Losappio, Nuccia Morici, Matteo Di Biase, Michele Nichelatti, Jan Walter Schroeder, Luca Balossi, Silvio Klugmann

**Affiliations:** The Department of Allergology and Immunology, Niguarda Ca’ Granda Hospital, Milan, Italy; The Department of Laboratory Medicine, Niguarda Ca’ Granda Hospital, Milan, Italy; The Department of Cardiology1-Emodinamic, Niguarda Ca’ Granda Hospital, Milan, Italy; The Department of Cardiology, University of Foggia, Foggia, Italy; Service of Biostatistics, Niguarda Ca’ Granda Hospital, Milan, Italy

**Keywords:** Tryptase, Acute coronary syndrome, Major adverse cardiovascular events, Biomarker, Mast cell

## Abstract

**Background:**

One of the greatest challenges in cardiovascular medicine is to define the best tools for performing an accurate risk stratification for the recurrence of ischemic events in acute coronary syndrome (ACS) patients.

**Methods:**

We followed 65 ACS patients enrolled in a previous pilot study for 2 years after being discharged, focusing on the occurrence of major adverse cardiovascular events (MACE).

The relationship between serum tryptase levels on admission, SYNergy between percutaneous coronary intervention with the TAXUS drug-eluting stent and the cardiac surgery score (SX-score), cardiovascular complexity and MACE at 2 years follow-up were analyzed.

**Results:**

The ACS population was divided in two groups: patients with MACE (n = 23) and patients without MACE (n = 42).

The tryptase measurement at admission (T0) and at discharge (T3) and SX-score were higher in patients who experienced MACE than in those without (p = 0.0001, p < 0.0001 and p = 0.006, respectively). Conversely, we found no significant association between MACE and C-reactive protein (CRP), and between MACE and maximum level of high-sensitivity troponin (hs-Tn) values.

Among all patients with MACE, 96% belonged to the group that presented with cardiovascular complexity at the beginning of ACS index admission (p < 0.0001).

The predictive accuracy of serum tryptase for MACE at follow up set at the cut-off point of 4.95 ng/ml at T0 and of 5.2 ng/ml at T3. Interestingly, patients with both the above cut-off tryptase values at T0 and at T3 presented a 1320% increase in the odds of developing MACE (p < 0.0001).

**Conclusion:**

In ACS patients, serum tryptase measured during index admission is significantly correlated to the development of MACE up to 2 years, demonstrating a possible long-term prognostic role of this biomarker.

**Electronic supplementary material:**

The online version of this article (doi:10.1186/s12948-015-0013-0) contains supplementary material, which is available to authorized users.

## Background

Major adverse cardiovascular events, such as myocardial infarction, cardiac arrhythmia, target vessel failure, heart failure, and death from all causes, are severe and feared sequelae of patients who develop ACS. Thus, one of the greatest challenges in cardiovascular medicine is to find a way to predict the risk of short- and long-term MACE in ACS patients.

During the last few decades, several proteins have been investigated as potential diagnostic and prognostic biomarkers in ACS patients. Indeed, atherosclerosis and plaque destabilization are due to a heterogeneous process involving vascular inflammation, endothelial dysfunction, and hypercoagulability. Some blood biomarkers may reflect the severity of these pathological processes associated with the progression of atheromatous lesion [1] and consequently predict the occurrence of MACE [2]. However, studies evaluating the prognostic role of several relevant biomarkers have not yet given conclusive and convincing results. Hs-Tn has shown an excellent negative prognostic value for predicting long-term MACE. Furthermore, hs-Tn is the reference standard for early diagnosis of myocardial infarction. However, to achieve the best clinical use, hs-Tn has to be used in conjunction with clinical judgment and ECG.

Indeed, hs-Tn increase may be an expression of plaque rupture, atherosclerosis or discrepancy between elevated oxygen demand and supply. Patients with plaque rupture do certainly profit from more aggressive antiplatelet therapy, whereas patients with atherosclerosis should be treated with a more conservative approach. Hs-Tn may also detect myocardial injury even without an ongoing ACS [3-5]. Therefore, to increase the performance of this biomarker for the diagnostic and prognostic purposes, we have to carefully consider the prevalence of disease within a given population and patient comorbidities. All these considerations warrant cardiovascular research to find a new and more meaningful biomarker. Evidence that chronic inflammatory states can aggravate atherogenesis are accumulating. Consequently, inflammatory biomarkers could be a great resource by helping to clarify the pathophysiological mechanisms linking inflammation to cardiovascular disease, to improve the prognostic value of existing tools, and to identify novel therapeutic targets. In this light, Pastorello et al. have recently evaluated the role of tryptase as a prognostic marker of cardiovascular complexity in ACS [6]. The results showed an association among this inflammatory biomarker and the angiographic extension of cardiovascular disease, as defined by the SX-score.

Tryptase is a mast cell serine protease mediator implicated in many different inflammatory processes. This protein might be involved in atherosclerotic plaque progression and destabilization in the ACS setting, and it could, thus, be a valid expression of the bulk of vascular involvement [7]. Therefore, tryptase could be a predictive marker of recurrent adverse events in ACS patients. The aims of this study were to evaluate the development of MACEs within 2 years after an initial coronary angiography in ACS patients and to define the relationship between serum tryptase levels, SX-score, and cardiovascular complexity reported during acute events, in order to understand the predictive role of serum tryptase levels.

## Methods

### Study population

A sample of 65 patients, consecutively admitted to the Cardiology Department from April 2012 to September 2012, who underwent coronary angiography for ACS was included in a previous study to evaluate the correlation between clinical data and tryptase levels. All the patients with a history of concomitant mastocytosis and severe allergic diseases were formally excluded. Furthermore, in the selected patients a clinical evaluation for autoimmunity, cancer, renal failure, myelodysplastic syndromes and hypereosinophilic syndrome or other hematological disorders was performed. The study subjects gave informed consent. These patients were evaluated for cardiovascular complexities based on the presence of at least two of the following clinical adverse events at hospitalization: at least two epicardial coronary arteries involved in the atherosclerotic disease, more than 1 stent implanted, or more than 2 coronary artery disease risk factors. In the cohort of 65 patients previously described, we performed a follow-up evaluation beginning at post-discharge and lasting 2 years.

This study was approved by the Ethical Committee of Niguarda Ca’ Granda Hospital (Protocol Registration System. ClinicalTrials.gov. Number:193_05/2012).

### Clinical follow-up

All the patients were assessed with regular controls and the follow-up data, focusing on the occurrence of MACE. Data were obtained from outpatient visits and medical records. MACE was defined as recurrent myocardial infarction, unplanned percutaneous coronary intervention (PCI), cardiac arrhythmias, systemic embolism, heart failure, and death from all causes. Myocardial infarction was defined as the clinical diagnosis of ST-elevation myocardial infarction (STEMI) and non-ST elevation myocardial infarction (NSTEMI) in accordance with current guidelines of the European Society of Cardiology [8]. Unplanned coronary revascularization was defined as unplanned repeated PCI or coronary bypass grafting. Cardiac arrhythmia was defined as any cardiac arrhythmia leading to prolonged hospitalization. Systemic embolism was defined as abrupt vascular insufficiency associated with clinical or radiological evidence of arterial occlusion in the absence of other likely mechanisms. Heart failure was defined as radiographic evidence of pulmonary edema, physical examination demonstrating pulmonary rales greater than basilar, the development of a new S3 gallop and the requirement for inotropic support or intravenous diuretic therapy for left-ventricular dysfunction. Death was defined as any cardiovascular and non-cardiovascular death occurring during the follow-up period.

### Biomarker evaluations

Details about the serum values of tryptase, cardiac troponin, and CRP measurement and their analyses were reported in a previous study [6]. Serum tryptase levels were measured at T0 and at T3 when all the symptoms and signs of the acute presentation were resolved. The quantitative measurement of tryptase was performed using an ImmunoCAP tryptase in vitro fluoro-enzyme-immunoassay test (Phadia, now Thermo Fisher Scientific, Uppsala, Sweden), according to the manufacturer’s instruction. This laboratory method measures the total tryptase levels of all proforms of α-tryptase and β-tryptase as well as mature β-tryptase. A normal tryptase level is considered < 5 ng/ml.

Cardiac troponin levels were the maximum value obtained, and the CRP levels were those obtained at admission.

### Statistical analysis

Data were analyzed by usual descriptive techniques, categorical data were tabulated with their relative and absolute frequencies, and continuous variables were described by the mean ± standard deviation, or by median and range.

Fisher’s exact test and the McNemar test were used to check significance of cross-tabulations, for between- and within-groups analyses.

Univariate and multivariate logistic regression were used to evaluate the effects of the variables on complication occurrence, with the Wald test used to check the significance of each single variable. For significant variables, a further ROC analysis was carried out to search for a possible cut-off value, and the corresponding ROC curve was traced. For these cut-off values, sensitivity, specificity, positive predictive value (PPV), and negative predictive value (NPV) were calculated.

## Results

The ACS population was divided in two groups: patients with MACE (n = 23; 35.3%: 15 STEMI and 8 NSTEMI) and patients without MACE (n = 42; 64.7%: 35 STEMI and 7 NSTEMI). In particular, among MACE patients, 16/23 have developed recurrent myocardial infarction, 3/23 systemic embolysm and 4/23 heart failure. Stent thrombosis occurred in 1 out of 16 patients with recurrent myocardial infarction. In patients with MACE, the tryptase values [median tryptase level at T0 7.02 ng/ml (range: 1.49 ng/ml to 31.6 ng/ml) and median tryptase level at T3 5.70 ng/ml (range: 1.60 ng/ml to 19.6 ng/ml)] were significantly higher than in those without MACE [median tryptase level at T0 4.55 ng/ml (range: 1.29 ng/ml to 13.6 ng/ml); median tryptase level at T3 3.80 ng/ml (range: 1.16 ng/ml to 11.3 ng/ml)].

Therefore, the association between high tryptase levels at T0 and T3 and subsequent development of MACE was statistically significant (Mann–Whitney U test: p < 0.0001) for the overall cohort. We found that any arbitrary unitary increase (+1 ng/ml) in tryptase values at T0 corresponded to a 38% [95% interval confidence (CI): 8% to 75%; p = 0.010] increase in the odds of developing MACE after two years from the acute event. At T3, we found that any arbitrary unitary increase (+1 ng/ml) in tryptase values corresponded to a 57% (95% CI: 13% to 118%; p = 0.008) increase in the odds of developing MACE after two years from the acute event.

**Figure 1:**
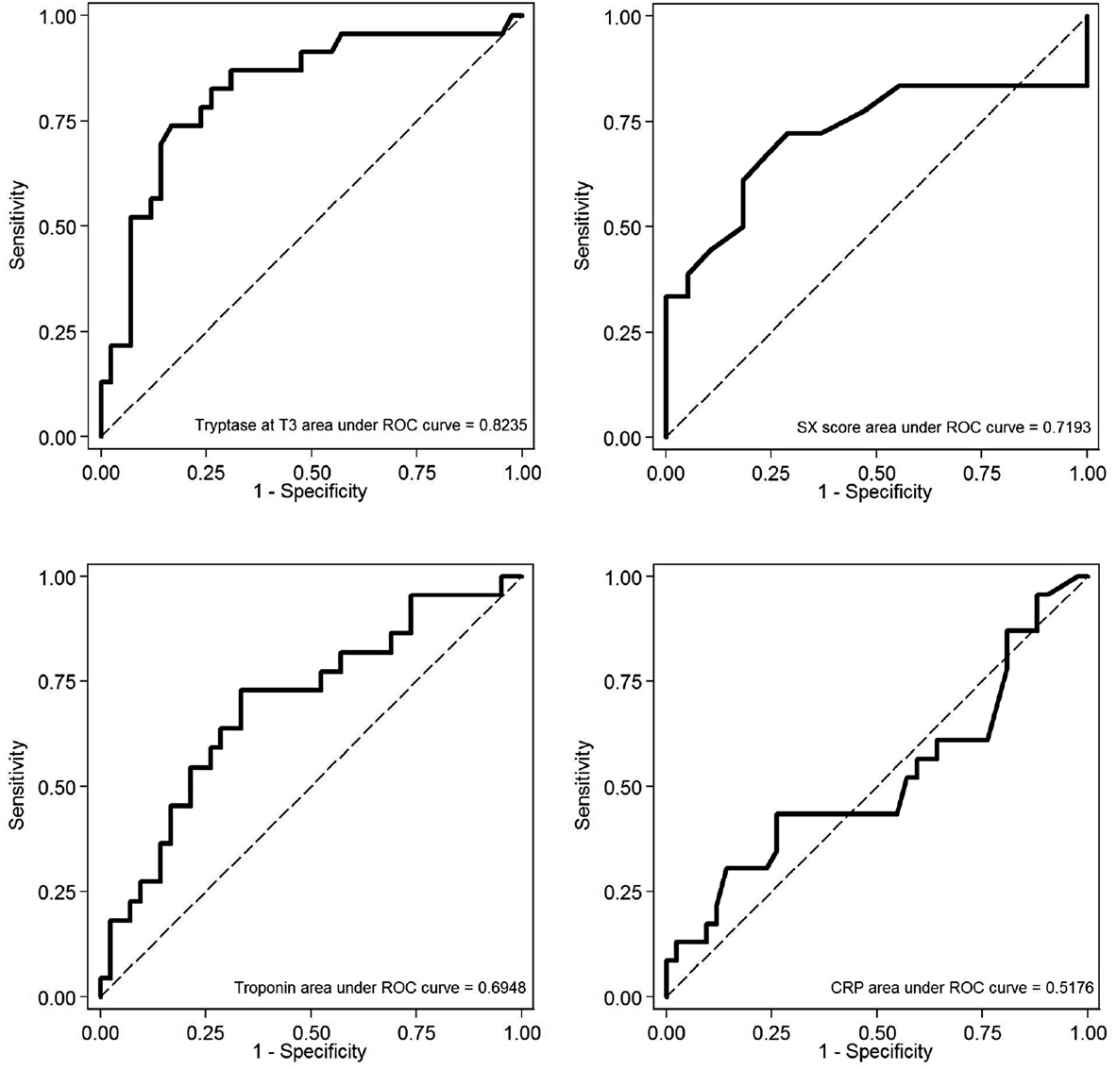
**ROC curves reporting AUC calculated at the time of acute event for tryptase,** **cardiac troponin**, **CRP, and SX-score, with respect to MACE: AUC of tryptase and SX-score appear significantly higher than the other AUCs.** ROC analysis to search for cut-off values of the different markers predictive of MACE.

We also found that, among all MACE patients, 96% belonged to the group that presented a cardiovascular complexity at the beginning of ACS index admission (McNemar test: p < 0.0001). The risk to develop MACE was also significantly correlated to cardiovascular complexity when adjusted for tryptase value at T0 and T3 (Wald test: p = 0.029, and p = 0.033, respectively). We searched for a possible cut-off value for tryptase values at T0 and at T3 as a predictor of MACE using the Youden method after a ROC analysis. The optimal cut-off for the tryptase at T0 was 4.85 ng/ml, where predictive positive value (PPV) and negative predictive value (NPV) were 94.9% (95%% CI 82.7% to 99.5%) and 76.9% (95% CI 56.4% to 91.0%), respectively, and with 86.0% sensitivity (95%% CI 72.1% to 94.7%) and 90.9% specificity (95% CI 70.8% to 98.9%). The optimal cut-off for the tryptase at T3 was 5.2 ng/ml (Figure 1), where PPV and NPV were 70.8% (95% CI 48.9% to 87.4%) and 85.4% (95% CI 70.8% to 94.4%), respectively, and with 73.9% sensitivity (95% CI 51.6% to 89.8%) and 83.3% specificity (95% CI 68.6% to 93.0%). Patients with tryptase value ≥ 4.85 ng/ml at T0 had a 700% increase in the odds of MACE development after 2 years (Wald test: p = 0.003). Tryptase value ≥ 5.2 ng/ml at T3 was associated with a 1300% increase in the odds of MACE development after 2 years (Wald test: p < 0.0001). Those with both the above cut-off tryptase values at T0 and at T3 presented a 1320% increase in the odds of developing MACE (Wald test: p < 0.0001).

**Figure 2:**
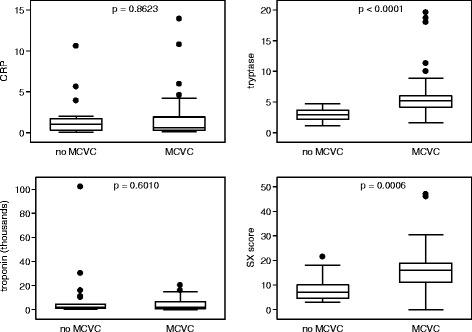
**Box plot for tryptase at T3, CRP, cardiac troponin measurements, and SX-score in MACE and non-MACE patients.** The p values were calculated by the Mann–Whitney test. Comparison of the measurements of the analysed markers in patients with and without MACE.

The boxplots in Figure 2 show the measures of association between MACE, serum tryptase at T3 and SX score: in both cases, values are significantly higher in those patients who developed MACE (Mann–Whitney U test: p < 0.0001 and p = 0.0006, respectively). Conversely, there was not relevant association between hs-Tn (p = 0.6010), nor CRP (p = 0.8623) and follow up MACE.

## Discussion

In the present study, we looked for the development of MACE at 2 years in a cohort of patients with ACS who were previously enrolled in a pilot study aimed at estimating the predictive value of serum tryptase [6]. The results demonstrated a significant relationship between the cardiovascular complexity and high tryptase levels during hospitalization. We also found that serum tryptase levels were significantly related to the severity of the coronary artery disease, as defined by the SXscore [9]. A total of 23 of 65 patients developed MACE after 2 years from the ACS, with 3 of them dying from cardiac causes and the others presenting a new myocardial infarction (n = 18) or systemic embolism (n = 2).

Therefore, in the present study, we analyzed the correlation between the development of MACE and some angiographic and clinical parameters, such as SX-score and cardiovascular complexity, and the previously evaluated biological biomarkers, such as serum tryptase, hs-Tn and CRP. This study demonstrated a correlation between a high SX-score and the development of MACE at two years. Patients with MACE demonstrated a mean SX-score of 16.8, compared to the measure of 9.2 found in patients without MACE. This result confirms the data in the literature. In a recent trial of 1,132 ACS patients undergoing PCI, patients who experienced MACE at 1 year presented high (≥19) or intermediate (11–18) SX-score [10]. This shows that the SX-score is a useful scoring system to assess the severity of coronary artery disease and to determine the appropriate revascularization strategy [11]. Furthermore, the SX-score is an independent predictor of MACE in ACS patients [12].

An original result of the present study was that 96% of MACE patients presented signs of cardiovascular complexity during the cardiac acute event, and only 1 out of 4 ACS complex patients did not develop MACE at 2 years, with a significant difference in cardiovascular complexity between patients with MACE and those without.

Thus, the presence of at least two signs of cardiovascular complexity upon admission to a hospital may be correlated to the recurrence of cardiovascular events.

Another finding was that a significantly higher level of serum tryptase was evident in patients with MACE compared to those without (mean tryptase levels of 13.8 ng/ml and 9.1 ng/ml, respectively. Conversely, when we analyzed serum cardiac troponin and CRP, no discriminative values were found among patients with or without follow-up events.

The ROC curves showed that the optimal cut-off values for serum tryptase in predicting high versus low MACE rate were ≥ 4.85 ng/ml and ≥ 5.2 ng/ml at T0 and at T3, respectively.

The correlation between high tryptase levels and the development of MACE could indicate that the mast cells have a role in the coronary atherosclerotic damage process, in which these cells contribute to plaque progression and destabilization. Bot et al. recently explained the potential roles of activated mast cells in the growth and destabilization of an atherosclerotic plaque [13]. Interestingly, one patient with tryptase level of 6.8 ng/ml experienced a very late stent thrombosis, that developed 18 months after the drug-eluting stent implantation. In this specific case, we cannot exclude a Kounis syndrome variant III, where drug-eluting stent components, including different metal strut, polymers or metal anions, may induce an antigenic complex responsible for a persistent inflammatory action [14-16]. On the other hand, as known, mast cells are activated by IgE, but also by IgE-independent mechanism, cytokines, chemokines, drugs, physical stimuli and hormones, therefore, these cells are not only involved in allergic diseases but also in inflammatory conditions such as atherosclerosis [17] as well as the chemotaxis of eosinophils and other inflammatory cells involved in thrombosis pathogenesis [16].

Atherosclerosis is an inflammatory disease characterized by the progressive accumulation of cholesterol in the intimal layer of arterial walls leading to the formation of plaque and vascular obstruction [18]. Mast cells, like other inflammatory cells, are located in the human arterial intima and adventitia [19], and when activated, they release granules locally, which contain a large panel of mediators, including neutral proteases (tryptase and chymase), cathepsins, heparin, histamine, cytokines, and growth factors. During early atherogenesis, the effector molecules stimulate leukocyte recruitment and lipid accumulation in the evolving plaque, whereas during advanced stages of atherogenesis, they contribute to the generation of an unstable plaque susceptible to rupture [13]. Tryptase activates metalloproteinases (MMP), such as MMP-1, MMP-2 and MMP-3 and procollagenase, and promotes the degradation of lipoproteins and fibronectin [20], acting as powerful inflammatory stimulus at the endothelial dysfunction [21,22]. Many experimental studies have confirmed the role of tryptase in the inflammatory process of atherosclerosis as well as in aortic aneurysm formation. Zhang et al. observed a reduction of abdominal aortic aneurysm formations in tryptase-deficient mice [23]. Wang et al. recently demonstrated that the absence, or pharmacological inhibition, of tryptase reduces abdominal aortic aneurysm formation in animal models [24].

On the basis of these observations, we can suggest that patients with high serum tryptase levels could have a greater burden of mast cells in the arterial wall. This burden contributes to the coronary lesion formation incidence of ACS and the recurrence of follow-up cardiovascular events.

## Conclusions

In conclusion, serum tryptase seems to be a non-invasive, promising prognostic long-term biomarker in ACS, STEMI and NSTEMI patients. Higher than 4.85 ng/ml tryptase levels at the onset of ACS were predictive for occurrence of MACE within 2 years from the acute event.

Further studies are necessary to define the precise pathophysiological role of tryptase in cardiovascular diseases and to confirm its role as prognostic biomarker of ACS.

## Availability of supporting data

Additional file 1 includes serum tryptase clinical data.

## Additional file

Additional file 1:
**Serum tryptase clinical data.**

## Abbreviations

ACS: Acute coronary syndrome
CI: Confidence interval
CRP: C-reactive protein
hs-Tn: Highsensitivity cardiac troponin
MMP: Metalloproteinase
NPV: Negative predictive value
NSTEMI: ST depression on ECG
PCI: Percutaneous coronary intervention
PPV: Positive predictive value
ROC: Receiver operating characteristic
STEMI: ST segment elevation on ECG
SX-score: SYNergy between percutaneous coronary intervention with the TAXUS drug-eluting stent and the cardiac surgery score
